# The Development and Evaluation of an Educational Video for Breast Cancer Patients Requiring Adjuvant Radiation Therapy

**DOI:** 10.1007/s13187-024-02408-x

**Published:** 2024-02-22

**Authors:** Yvonne Moussa, Yobelli Jimenez, Wei Wang, Najmun Nahar, Verity Ahern, Kirsty Stuart

**Affiliations:** 1 Sydney Medical School, C24–Westmead Hospital, The University of Sydney, Sydney, NSW Australia; 2 Discipline of Medical Imaging Science, Faculty of Medicine and Health, The University of Sydney, Sydney, NSW Australia; 3 Westmead Breast Cancer Institute, Westmead Hospital, Sydney, NSW Australia; 4 Department of Radiation Oncology, Crown Princess Mary Cancer Centre, Westmead Hospital, 166-174 Hawkesbury Rd, Westmead, NSW 2145 Australia; 5 Department of Radiation Oncology, Blacktown Cancer and Haematology Centre, Blacktown Hospital, Blacktown, NSW Australia

**Keywords:** Breast cancer, Radiotherapy, Patient education, Video education, VERT

## Abstract

Patient education and informed consent are required prior to adjuvant radiation therapy (RT) for early breast cancer (EBC), and include the role, rationale, potential toxicities and practicalities of the treatment process. Current education of patients about RT is verbal, in the form of a consultation by a radiation oncologist, often supplemented with print or online materials. This approach is limited by its doctor-dependency and non-standardised nature. Video education is being recognised increasingly as an opportunity to remediate this and appeal to patients’ preference for visual learning. The purpose of this study was to design and produce a video as an educational adjunct for EBC patients’ viewing prior to adjuvant RT, and to evaluate its acceptability through content analysis of three online focus group discussions, among ten participants with EBC requiring RT. After qualitative content analysis of the focus group transcripts, data were summarised into three main categories: (1) understanding of RT, (2) ease of engagement with the video and (3) anxiety and preparedness for RT. The 18-min video was positively received by all participants, and discussion feedback was used to inform improvements to the video. This focus group study demonstrated that the video was well understood, informative and acceptable to EBC patients in preparing them for RT. The effectiveness of the video in improving knowledge and alleviating distress in preparation for therapy will be further evaluated in an ethics-approved biphasic quasi-experimental study.

**Supplementary Information:**

The online version contains supplementary material available at 10.1007/s13187-024-02408-x.

## Introduction

Adjuvant radiation therapy (RT) after breast-conserving reduces the risk of both locoregional and distant recurrence and improves overall survival in women with early breast cancer (EBC) [1, 2]. The uptake of RT can be limited due to its perceived complexity and potential side effects, which warrants that patients attain a minimum baseline understanding of essential information to adequately consent and collaborate through their treatment [3]. Both historically and currently, the most common form of RT education is one-on-one verbal communication with the radiation oncologist (RO) during the initial consultation and is often reinforced with print and occasionally online materials [4]. Surveys of Australian EBC patients have identified informational needs to be highest during the initial RO consultation, where the patient is educated on potential benefits and risks of RT, to provide adequate informed consent [5, 6]. However, verbal education has limitations, including that it is doctor-dependent and non-standardised in quality and depth [4]. Similarly, print and online information is often non-individualised and poorly understood in the presence of language barriers or low health literacy [7, 8]. There has been a rapid expansion of internet-based information wherein breast cancer is the most frequently researched cancer [9], yet a substantial proportion of online information may be inaccurate or irrelevant [5].

Despite the growing importance assigned to RT education in the literature, substantial numbers of EBC patients continue to report unmet informational needs and suboptimal treatment knowledge, particularly relating to side effects of RT [5, 10]. Additionally, the mechanism underpinning RT is not generally familiar to patients, as radiation is a complex concept with limited visual or sensory stimuli [11], so patients may struggle to conceptualise RT or conflate its use with perceived harms [12]. Traditional verbal and print material is often inadequate in capturing this, giving rise to an appetite for modalities that appeal to preferences for visual learning and extend patient education beyond merely a verbal discussion and generic written material [4]. The evolving area of breast cancer RT education literature is increasingly recognising video technology as an opportunity to remediate the limitations of current educational modalities [7, 8, 13, 14].

The objective of this study was to design and produce an educational video to meet the informational needs of EBC patients commencing adjuvant RT at a metropolitan tertiary teaching hospital. Subsequently, the study aimed to evaluate acceptability of the video through content analysis of focus group discussions, to inform improvements to the video.

## Methods

### Study Overview

This study involved the design and production of a video as an educational adjunct for EBC patients prior to commencing adjuvant RT, with the view to replace part of the standard verbal education delivered during the initial RO consultation. The video underwent qualitative evaluation by content analysis of three focus group discussions among EBC patients commencing RT. Study approval was obtained from the Western Sydney Local Health District (WSLHD) Human Research Ethics Committee (Number 2021: ETH12077).

### Video Development

**Fig. 1:**
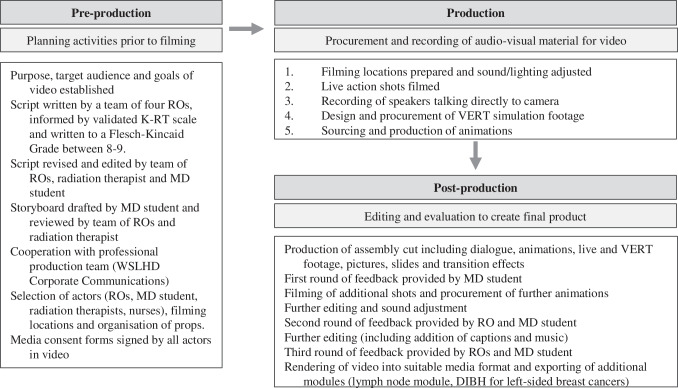
Summary of steps in pre-production, production and post-production of video. Abbreviations: RO, radiation oncologist; K-RT, Knowledge of Radiation Therapy; MD, Doctor of Medicine; WSLHD, Western Sydney Local Health District; VERT, Virtual Environment for Radiotherapy Training; DIBH, Deep Inspiration Breath Hold

Video development occurred in three phases: pre-production, production and post-production, summarised in Fig. 1. To maximise authenticity and familiarity, all staff and filming locations were within the Westmead Breast Cancer Institute (WBCI) or Crown Princess Mary Cancer Centre Westmead (CPMCCW). Production and post-production services were completed by the WSLHD Corporate Communications. Simulations of RT apparatuses and imperceptible concepts such as radiation and internal patient anatomy were procured using Virtual Environment for Radiotherapy Training (VERT) software (version 2.9) [15]. The video covered the 30-item Knowledge of RT scale identified as seminal by the “RT Prepare” study [16]. Table 1 provides an overview of the 18-min video.

**Table 1:** Description of structure and content of educational video

Segment	Content		Visual stimulus
Introduction	- Greeting by RO and brief introduction to concepts covered in the video		RO speaking to camera
What is RT?	- Establishment of RT as a safe and effective treatment delivered by RO team - Explanation of mechanism by which RT targets cancerous cells, while normal healthy cells are capable of repair		RO speaking to camera, overlayed: - Live shots of linear accelerator - Animation of cell repair - VERT simulation footage
What is involved in having RT?	CT planning	- Detailed explanation of CT-planning session, including patient positioning, orientating tattoos imprinted onto the skin, scans performed and the subsequent planning processes performed by radiation therapists, ROs and physicists	RO speaking to camera, overlayed with: - Live shots of patient during planning session and radiation therapists planning treatment
	Daily treatment	- Practicalities of receiving RT treatment, including days on which treatment is provided, duration of process, parking and accessibility - Detailed explanation of RT treatment process, including positioning, duration and RO reviews during treatment - Debunking of myths around receiving RT (e.g. radioactivity of patient is false) - Precautions such as avoiding pregnancy	Radiation therapist and RO speaking to camera, overlayed: - Live shots of RT treatment process and RO reviews - Animations
Side Effects Acute and Longer-term	- Overview of acute and longer-term side effects of fatigue, skin reactions, nausea, breast swelling and tenderness, including management of side effects (if required)		NUM speaking to camera, overlayed: - Live shots of NUM examining skin
Late Side Effects Uncommon	- Overview of uncommon late side effects, including prolonged skin reactions (e.g. permanent darkening, pale patches, prominent blood vessels), residual breast swelling, changes to shape and size of the breast and local rib/muscle discomfort		RO speaking to camera
Late Side Effects Rare	- Overview of rare late side effects such as spontaneous rib fractures, radiation pneumonitis, heart disease and future cancers - Explanation of risk minimisation strategies during delivery of RT (i.e. deep inspiratory breath hold for heart disease in left-sided breast cancers) and general measures (e.g. smoking cessation to reduce risk of future cancers, cardiovascular risk reduction for heart disease)		RO speaking to camera, overlayed: - Live shots of ROs discussing a CT scan - Animations - VERT simulation footage
Lymph Node RT Supraclavicular fossa ± Axillary	- Additional module for patients with lymph node involvement - Explanation of rationale for treating lymph nodes and overview of additional rare side effects, such as lymphoedema and brachial plexus neuropathy, including management of these side effects		RO speaking to camera, overlayed: - Live shots of RO reviews - Animations - VERT simulation footage
Conclusion	- Invitation to discuss specific questions and concerns with RO		RO speaking to camera

*RT*, radiation therapy; *RO*, radiation oncologist; *NUM*, nursing unit manager; *VERT*, Virtual Environment for Radiotherapy Training

### Participant Recruitment

Convenience sampling was used to recruit EBC patients presenting to WBCI or CPMCCW between March and June 2022 for their initial RO consultation, into three focus groups of three to four participants each. Eligible women were aged > 18 years, with sufficient English language proficiency, hearing and vision, diagnosed with unilateral EBC and scheduled to receive standard adjuvant RT in 15–25 fractions after breast-conserving surgery. Patients with a prior history of RT, mastectomy, breast reconstruction, significant active psychiatric disorder or who were pregnant, lactating or deemed unsuitable by their clinician were ineligible. Eligible patients were provided information by their Breast RO and invited to participate, after RT consent but prior to RT commencement. Ethics approval included trial consent on day of RT consent, provided sufficient time was allowed for trial consideration, or later, prior to RT. Participants with written informed trial consent viewed an online screening of the video, immediately prior to focus group discussions, which were held via Zoom due to COVID-19.

### Data Collection

Immediately after providing informed consent, trial participants were asked to complete a brief questionnaire to obtain demographic information and quantify their level of distress using the National Comprehensive Cancer Network’s Distress Thermometer. Clinical data about their EBC, treatment regimen and Eastern Co-operative Oncology Group (ECOG) performance status, were collected from medical records. For staging of EBC, the American Joint Committee on Cancer’s Tumour, Node, Metastases (TNM) system was used. All participant data were de-identified and input into Excel™. Every three or four participants were grouped in order of recruitment into one of three focus groups. The approximately 30-min-long focus group discussions were facilitated and audio-recorded via Zoom. The discussion was moderated by author YM, supervised by YJ, with prompts stimulating reflection on video effectiveness pertaining to understanding of RT, anxiety, preparedness and ease of engagement. Audio recordings were transcribed verbatim.

### Data Analysis

Demographic data were descriptively grouped into categories. Excel™ was used to produce descriptive statistics to represent participant data as percentages and means. Once accurate transcriptions of focus groups were confirmed, they were uploaded onto NVivo (QSR-International), a qualitative data management and coding software. Digital recordings were deleted. Data were analysed by content analysis, wherein transcripts were reviewed line-by-line by two authors independently, generating initial codes that were then further synthesised into meaningful code categories. The final analysis was completed by two authors (YM, YJ) independently and discussed until consensus reached.

## Results

### Participants

Ten women participated in three focus groups between May and July 2022, with four participants in the initial group, and three in both latter groups. Mean age of the women was 62 years (range 43–74 years). Most women were married or in a de-facto relationship (90%), and all resided within surrounding suburbs of our metropolitan hospital. While all participants were English-speaking, 30% spoke an additional language at home and 40% were born overseas. Employment statuses included full-time (30%), part-time (10%), retired (30%) and other work (30%), including volunteering and homemaker roles. The highest level of education attained ranged from school certificate or diploma qualifications (60%) to higher school certificate (20%) and university education (20%). All women were ECOG 0–1. The participants’ EBCs were left-sided for 70%, while 30% were right-sided. The majority of tumours were T1 (60%), with the remainder T2 (20%) and Tis (20%); all tumours were N0. Additionally, 70% of women reported low distress scores of ≤ 3, and 30% reported distress scores ≥ 4. None received chemotherapy. Participant demographic and clinical data are summarised (Online Resource 1).

### Focus Group Findings

After analysis of the focus group transcripts, codes emerged under three main categories: (1) understanding of RT, (2) ease of engagement with video and (3) anxiety and preparedness for RT. These categories, as well as the codes within them and supportive direct quotes, are presented in Table 2.

**Table 2:** Key codes arising from focus group discussions and illustrative supportive quotes

Code	Quotes
Understanding of RT	
Improved understanding and expectations	“I wish I had seen it before I started, I would have understood more because I had no idea what to expect.” *P1* “This video is very detailed, and it takes you through the step-by-step procedure of what to expect.” *P2* “It doesn’t really matter what somebody tells you is going to happen, to see it really helps.” *P8* “You can understand more by seeing what you will be going through.” *P9*
Learnt new information about RT	“I had no idea when I started about the machine… I didn't know how big the machines were and that they had to be so precise with the lining up, even though it had been explained.” *P1* “One of the side effects is feeling nausea, I didn’t realise that.” *P1* “I’m watching for the first time how the whole machine is moving actually.”* P5*
Clarity of video content	“I think that if they explained a little bit more about the day off being the machine being serviced, a little bit more detail into that it would have been easier to understand.” *P1* “I understood the video quite clearly and it all made sense to me.” *P3* “The person who spoke about the length of time, the 15 min every day. She just said something about Monday or Friday that I didn't understand. I didn't get it quick enough.”* P4* "The information is very clear, nothing is lacking. It’s a very useful video and easy to understand.” *P5*
Complemented other information sources	“This video is like a graphic representation of the brochure. It’s more of a deeper explanation and then your doctor will explain it even further. It complements all the literature and explanations that were given to you.” *P2* “I found that I had already been presented with most of the information but at the time it was at the end of a five hour wait so I wasn’t particularly receptive to hearing anything. I read all the brochures given to me and did my own research online. If we could have seen this, it would have saved me a lot of time.” *P8*
Ease of engagement with video	
Usefulness of graphics in representing concepts	“I think because they showed the graphics when it got to the lymph nodes and it pointed to where it was, that made it much easier to understand.” *P1* “The graphics about the cancer cells, it’s a bit like watching the COVID germs jump around on the TV every night. I thought it was really good, better than the verbal education that I got.” *P8* “Animation is the key now to show people, I’m a visual learner.” *P9*
Appropriateness of video duration and pace	“I think to be able to compress everything, all the steps, expectations, processes, side effects, supports, the after-effects, all the possibilities in the 18–20 min video was really good.”* P2* “Many speakers were very articulate and easy to understand, not slow but well-paced but there are some speakers who were kind of abrupt and fast and so I had to pace myself in understanding.” *P2* “I think it was just perfect…it just explained everything so well and I really don’t think it needed to be any longer. If it was any shorter, maybe we might have missed something.” *P4* “If I could watch it two or three times, then I would cover everything.” *P8*
Appropriateness of vocabulary	“The language was very easy to understand.”* P5* “In layman’s terms, very good…It wasn’t too technical or scientific. For the most, you could understand everything they were saying. So from that point of view, I thought it was good.” *P10*
Anxiety and preparedness for RT	
Reduced anxiety towards RT	“If I’d seen the video first, I was at ease anyway, but I would have felt much more at ease.” *P1* “The speakers speak with calmness… an important factor for me is the calmness, it has to be maintained throughout the whole video in my opinion because you’re talking about radiotherapy and treatment.” *P2* “It makes you more relaxed a little bit knowing what is going to happen and gives us more confidence.” *P7*
Increased feelings of preparedness	“I am confident that if I had seen these videos before, I would be very confident.” *P5* “I like the fact that it felt so familiar. It just felt reassuring to see all that. I think had I seen that before I went it would have made me a little bit more comfortable on the day, knowing what I was going to have done and where it was going to be having a familiarity about.”* P10*
Changes in levels of concern regarding RT	“Unchanged because it was explained to me very well in one of my appointments.” *P1* “I think the side effects are more concerning, it’s clearly mentioned so we know what they are going to be.” *P5* “I will be the same. I will be prepared but I still am having a little bit of worries and am scared.” *P7* “I would say slightly decreased hearing about the after effects, part of it was reassuring.”*P10*
Unanswered questions remaining around RT	“What if we get COVID? What happens then?” *P1* “It was mentioned during the video that they don’t know at the time of your radiation therapy whether or not this has actually worked in eliminating any potential cancer cells that may or may not still be there. My next appointment with my doctor is in 12 months… how am I going to know whether or not the radiation therapy has worked?” *P8* “It may be helpful if there was something in the video about the percentages that I think were quoted at me originally about successful treatment with radiation versus not having any radiation.” *P10*

#### Understanding of RT

The category of understanding of RT included four main codes: improved understanding and expectations, learning new information, the video as a complement to other information sources and clarity of video content. Although most participants acknowledged they had some understanding of RT after their initial RO consultation, viewing the video enhanced most women’s understanding of RT. In particular, participants found the level of detail and step-by-step explanation improved understanding and provided expectations. There was also an appreciation of the ability to visualise the size and motion of the linear accelerator. Most participants found the video content to be clear, but two participants raised that the explanation of the typical weekly RT schedule and machine service was fast-paced and not articulated clearly. While some participants felt the video alone could have replaced hours of independent research and conversation with the RO, most women appreciated the video as a complement to other educational modalities.

#### Ease of Engagement with Video

The category of ease of engagement with the video included three main codes: usefulness of graphics in representing concepts, appropriateness of video duration and pace and appropriateness of vocabulary. All participants appreciated the incorporation of graphics, with some women attributing this to the appeal of visual modes of learning. Participants were satisfied with the length and thoroughness of the video. However, while most felt the video was appropriately paced, some expressed that parts were fast-paced and would benefit from watching the video multiple times. All participants felt that the video language was understandable and pitched at an appropriate level.

#### Anxiety and Preparedness for RT

Feedback regarding the effect of the video on anxiety and preparedness for RT included four codes of reduced anxiety towards RT, increased feelings of preparedness, changes in level of concern regarding RT and unanswered questions remaining around RT. Participants expressed that the video reduced their anxiety and made them feel increasingly prepared for RT. Some women who had already commenced RT expressed that seeing the video previously would have reduced their apprehension. After viewing the video, most participants felt their level of concern regarding RT had decreased or remained unchanged; one participant found the listing of side effects made her more concerned about starting RT. While most women felt the video comprehensively answered their questions, some women expressed uncertainty around contracting COVID-19 during treatment, consequences of missed RT sessions, differences in completing RT at other WSLHD hospitals and what follow-up and imaging are indicated after treatment completion.

#### General Positive and Other Reactions to Video

Among the three focus group discussions, the video was positively received by all ten participants, and all would recommend it to other women commencing RT. The focus groups suggested improvements, such as incorporating statistics on RT efficacy. Also, with the volume of information being provided, participants felt it would be beneficial to have external access to the video and share it with family members.

## Discussion

This study qualitatively evaluated the acceptability and effectiveness of a breast cancer RT video designed to educate and facilitate informed consent during the initial RO consultation. The video modality provides an approach to standardising depth and quality of the plethora of information that EBC patients encounter. Reception was largely positive with regard to ease of engagement with the video, its ability to improve understanding of RT and increase feelings of preparedness. The multi-modal incorporation of visual, auditory and written stimuli within the video appealed to participants, allowing the portrayal of abstract concepts such as radiation. To accommodate the low health literacy noted in 60% of Australian adults, the video was tailored to an eighth-grade level [17]. The video also balanced comprehensiveness and succinctness, with its duration within the typical range documented in the literature of 16–25 min, and being shorter than the standard RO verbal consultation of up to 40 min, amongst the practice of the RO authors.

The positive reception to this video is consistent with the growing body of evidence supporting video education in breast cancer RT education literature. Several recent studies have demonstrated educational videos to be a superior form of breast cancer RT education, leading to substantial improvements in patient self-reported knowledge [7, 8, 13], post-consult anxiety [7, 8, 13, 14], heightened confidence in providers [7] and better decision-making [7]. However, of these studies, only one single-arm study of 20 participants targeted the initial RO consult and reported the 7.5-min video to be a feasible intervention that decreased patient-reported anxiety [14]. Our study is the first to incorporate *VERT* simulations into a breast cancer RT educational video that is intended to be viewed by patients during an initial RO consultation. VERT-based education sessions have reliably demonstrated improvement of RT knowledge in EBC patients [18].

Although the majority of feedback was positive in nature, the discussions generated several insightful suggestions prompting the team to consider minor enhancements to optimise the video. These include subtitling the video to improve understandability, translating versions, and creating alternative versions with accessibility information for other WSLHD hospitals. Other suggestions were difficult to action, such as addressing the effect of COVID-19 on RT treatment despite being valid and pertinent, as policies and procedures are frequently changing. As such, it would be optimal to seek specific advice based on contemporary local policy. Similarly, patient-specific questions on follow-up imaging after RT completion or statistical recurrence risk data would be better suited to discussion with the RO after viewing the video.

There were challenges and limitations encountered in this study, some of which are inherent to focus group methodology. One potential limitation is that of volunteer bias, wherein volunteers may be systematically different to the general breast cancer population [19]. This may have further been compounded by the inclusion criteria for English proficiency. However, this study captured women across a range of ages, educational levels and linguistic backgrounds. Similarly, the phenomenon of group bias arises in focus groups, wherein participants may opt to maintain group consensus rather than present their true opinion. Overall, the focus group methodology was conducive to the aim of gathering preliminary feedback on the newly created video. Although a face-to-face format would have been preferred due to non-verbal communication and visual cues [20], Zoom emerged as a non-inferior alternative during COVID-19 [21]. The pandemic also saw delays in participant recruitment, which limited this study to three focus groups. The smaller groups not only facilitated equal discussion opportunities among participants, but are substantiated by previous work demonstrating 80% of discussion themes are discoverable within two to three focus groups [22]. Another challenge was creating a video specific enough to educate and answer patient questions but general enough to maintain relevance to most patients. Hence, two additional video modules were created to be integrated into the core video for specific patient groups with lymph node involvement or left-sided cancers.

In the future, the video will be further evaluated within an ethics-approved biphasic quasi-experimental pilot trial. The trial will assess knowledge of RT and evaluate levels of anxiety and distress in EBC patients, who will receive at the initial RO consultation either standard education, or have *part* of the standard education replaced by this video in addition to the residual standard education and individual patient-specific information. If benefit from video education is demonstrated in the pilot trial over standard practice, this research has the potential to enhance standard EBC RT education at the initial RO consultation. Multimedia-based interventions are cost-effective [23], time-efficient [24] and allow the delivery of high-quality education remotely. Further efforts will be required to extend the generalisability of this video to include patients from culturally and linguistically diverse backgrounds, and other health areas.

## Conclusions

An educational video was created for EBC patients prior to commencing RT, to replace part of the standard verbal education offered during the initial RO consultation, to be followed immediately by an individualised discussion with the RO. This video was qualitatively reviewed with content analysis after focus group discussions within this population. The video was positively received in terms of engagement, ability to improve understanding, alleviate anxiety and increase preparedness for RT.

### Supplementary Information

Below is the link to the electronic supplementary material.

Supplementary file1 (DOCX 32 KB)

## Data Availability

Research data are not available at this time.

## Code Availability

No.
