# Supplementary material for: The Development and Evaluation of an Educational Video for Breast Cancer Patients Requiring Adjuvant Radiation Therapy
Source: J Cancer Educ. 2024 Feb 22;39(3):279–87. doi: 10.1007/s13187-024-02408-x (PMC11102385; doi:10.1007/s13187-024-02408-x)
Supplement: Supplementary file 1 — Supplementary file1 (DOCX 32 KB) [file 13187_2024_2408_MOESM1_ESM.docx]

**Online Resource 1**

**Article title:** The development and evaluation of an educational video for breast cancer patients requiring adjuvant radiation therapy

**Journal:** Journal of Cancer Education

**Author names:** Yvonne Moussa, Yobelli Jimenez, Wei Wang, Najmun Nahar, Verity Ahern, Kirsty Stuart

**Corresponding Author:** Associate Professor Kirsty Stuart

Affiliations:

Sydney Medical School, C24–Westmead Hospital, The University of Sydney, Sydney, New South Wales, Australia

Westmead Breast Cancer Institute, Westmead Hospital, Sydney, New South Wales, Australia

Department of Radiation Oncology, Crown Princess Mary Cancer Centre, Westmead Hospital, NSW, Australia

Email: [Kirsty.Stuart@health.nsw.gov.au](about:blank)

**Supplementary Table 1** Demographic and clinical characteristics of focus group participants

| **Characteristic** | **Number of participants (%)**  (n=10) |
| --- | --- |
| Female sex | 10 (100) |
| Age group (years) |  |
| 40-49 | 1 (10) |
| 50-59 | 3 (30) |
| 60-69 | 4 (40) |
| 70-79 | 2 (20) |
| Highest level of educational attainment |  |
| University education and above | 2 (20) |
| Higher school certificate | 2 (20) |
| Diploma/apprenticeship | 5 (50) |
| School/intermediate certificate | 1 (10) |
| Employment status |  |
| Full-time employment | 3 (30) |
| Part-time employment | 1 (10) |
| Retired | 3 (30) |
| Other* | 3 (30) |
| Country of Birth |  |
| Australia | 6 (60) |
| Other | 4 (40) |
| Languages other than English spoken at home |  |
| Spanish | 1 (10) |
| Persian  Indo-Aryan | 1 (10)  1 (10) |
| Marital status |  |
| Married/De-facto relationship | 9 (90) |
| Not married† | 1 (10) |
| ECOG†† performance status |  |
| 0 | 3 (30) |
| 1 | 7 (70) |
| Tumour site |  |
| Right | 3 (30) |
| Left | 7 (70) |
| Tumour staging (as per TNM) |  |
| Tis | 2 (20) |
| T1 | 6 (60) |
| T2 | 2 (20) |
| Lymph node involvement | 0 (0) |
| Distress thermometer score |  |
| 0-1 | 3 (30) |
| 2-3 | 4 (40) |
| 4-5 | 1 (10) |
| 6+ § | 2 (20) |

Symbols:

* Category includes volunteer and homemaker

† Category includes single, divorced, separated, widowed

†† ECOG, Eastern Cooperative Oncology Group

§ Offered psychological review
